# Microalgal and Nitrogen-Fixing Bacterial Consortia: From Interaction to Biotechnological Potential

**DOI:** 10.3390/plants12132476

**Published:** 2023-06-28

**Authors:** Angel Llamas, Esperanza Leon-Miranda, Manuel Tejada-Jimenez

**Affiliations:** Department of Biochemistry and Molecular Biology, Campus de Rabanales and Campus Internacional de Excelencia Agroalimentario (CeiA3), Edificio Severo Ochoa, University of Córdoba, 14071 Córdoba, Spain; q72lemie@uco.es (E.L.-M.); q62tejim@uco.es (M.T.-J.)

**Keywords:** microalgae, diazotroph, nitrogen fixation, consortia, mutualism, biotechnology

## Abstract

Microalgae are used in various biotechnological processes, such as biofuel production due to their high biomass yields, agriculture as biofertilizers, production of high-value-added products, decontamination of wastewater, or as biological models for carbon sequestration. The number of these biotechnological applications is increasing, and as such, any advances that contribute to reducing costs and increasing economic profitability can have a significant impact. Nitrogen fixing organisms, often called diazotroph, also have great biotechnological potential, mainly in agriculture as an alternative to chemical fertilizers. Microbial consortia typically perform more complex tasks than monocultures and can execute functions that are challenging or even impossible for individual strains or species. Interestingly, microalgae and diazotrophic organisms are capable to embrace different types of symbiotic associations. Certain corals and lichens exhibit this symbiotic relationship in nature, which enhances their fitness. However, this relationship can also be artificially created in laboratory conditions with the objective of enhancing some of the biotechnological processes that each organism carries out independently. As a result, the utilization of microalgae and diazotrophic organisms in consortia is garnering significant interest as a potential alternative for reducing production costs and increasing yields of microalgae biomass, as well as for producing derived products and serving biotechnological purposes. This review makes an effort to examine the associations of microalgae and diazotrophic organisms, with the aim of highlighting the potential of these associations in improving various biotechnological processes.

## 1. Introduction

Algae are defined as a highly diverse group of eukaryotic organisms that undergo photosynthesis, but lack true roots, stems, leaves, and other specialized multicellular structures found in higher plants. Algae are considered one of the largest primary producers on our planet [1]. Within the algae group, the unicellular ones are referred to as microalgae. Although primarily abundant in aquatic ecosystems, microalgae can also thrive in various other habitats, such as terrestrial environments, within the crowded rhizosphere [2], in lichens [3], and even inside animal as in coral reefs [4]. Additionally, microalgae have been found on glaciers and ice sheets [5]. Microalgae exhibit a wide range of architectures, morphologies, and cell sizes. Their lifestyles are highly diverse and can be categorized as photoautotrophic, heterotrophic, or mixotrophic. Interestingly, microalgae have a broad metabolic capacity, some of which are unique and thus attract scientific research attention [6]. 

Symbiosis is a phenomenon widely utilized in nature, taking on multiple forms and serving various objectives that can be beneficial for only one of the organisms (commensalism, parasitism) or for both (mutualism) [7]. It has been observed that microalgae are capable of establishing symbiotic interactions with a diverse range of organisms, including plants, animals, fungi, and bacteria [8,9]. The term “phycosphere” refers to the area beyond the microalgal cell membrane, including the extracellular aqueous zone where bacteria interact with the microalgae. This region has no precise boundary and can extend to different distances around the microalgae [10]. In this area, bacterial growth is stimulated by the extracellular products of the microalgae, and vice versa. However, negative interactions between microalgae and bacteria mediated by various secondary metabolites (phycotoxins) have also been described [11]. In order to understand the interactions between microalgae and bacteria, several studies have focused on characterizing the microbiota of the phycosphere. It has been observed that the bacterial communities in the phycosphere are taxonomically distinct from those present in environments without microalgae [12]. Microalgae release significant amounts of dissolved organic matter that accumulates in the phycosphere, and these molecules are utilized as food or energy by the organisms that inhabit this niche [13]. Generally, the microalgae provide their partners with oxygen and a carbon source fixed through photosynthesis (photosynthates). In exchange, the microalgae receive some molecules necessary for their survival in their habitat, usually vitamins, hormones or a source of nitrogen [14,15]. The molecular and taxonomic diversity of mutualistic interactions between microalgae and their partners is significant. It is worth noting that the ability of organisms to adapt through mutations plays a critical role in the establishment of these interactions [16]. 

Nitrogen (N) is abundant in the atmosphere in the form of a dinitrogen gas (N_2_), but only a small number of prokaryotic organisms, known as diazotrophs, are capable of using it directly as a nitrogen source through biological nitrogen fixation (BNF) [17]. BNF is a highly regulated process due to the high energetic demands of the main enzyme involved in this reaction, nitrogenase. Nitrogenase requires 16 moles of ATP and 8 moles of electrons to reduce one mole of N_2_ to ammonium [18]. Several organisms rely directly or indirectly on diazotrophs and establish symbiotic interactions with them for proper growth. One of the most extensively studied interactions is the endosymbiotic relationship established between the roots of legumes and diazotrophic organisms called rhizobia, which provide fixed nitrogen to plants in exchange for protection, nutrients, and energy [19]. Mimicking this natural process, various diazotrophic organisms are also being tested as biofertilizers in non-leguminous plants [20]. The phycosphere of different green microalgae with great potential in the industry as *Chlamydomonas reinhardtii* (hereafter *Chlamydomonas*) [21], *Chlorella vulgaris* [22], *Scenedesmus quadricauda* [23] and *Botryococcus braunii* [24] has been characterized, and interestingly, some of them contain diazotrophic organisms such as *Rhizobium* sp. [21,24]. 

Over the past few years, there has been an exponential increase in the use of microalgae for various biotechnological applications [25]. Microalgae are crucial to sustaining the ecosystems, however occasionally they also produce harmful effects, like algal blooms that can cause a great ecological, economical and health impact [26]. In recent years, several studies have evaluated the role of naturally occurring diazotrophic association in organisms where microalgae play an essential function, such as corals and lichens. Additionally, studies have shown that the use of microalgae and diazotrophic organisms in consortia is very convenient for certain biotechnological applications.

In this review, we aim to gather the main available information on the different interactions studied between microalgae and N-fixing bacteria. Firstly, we present the interactions of microalgae with non-photosynthetic diazotrophic organisms, with various species of *Azotobacter* and *Azospirillum* standing out. Next, we move on to photosynthetic diazotrophic organisms, primarily focusing on cyanobacteria. Studies conducted with corals and lichens are also presented, where in some cases, the interactions between diazotrophs and microalgae also seem to play an important role. At the end, studies on different types of biotechnological applications utilizing the microalgae and N-fixing bacteria consortium are gathered and presented. The main objective is to draw attention to the fact that optimizing these consortia can lead to a significant decrease in the economic costs of cultivating these microorganisms.

## 2. Microalgae Interaction with Diazotrophic Bacteria

Plant growth-promoting bacteria (PGPB) enhance plant growth through various mechanisms, mainly by improving nutrient absorption in the soil. These bacteria have the ability to solubilize phosphorus, fix nitrogen, synthesize siderophores, produce phytostimulant substances such as auxins, gibberellins, cytokinins, and act as stress controllers in plants, among others [27]. An analogous concept, although less studied, is that of microalgae growth-promoting bacteria (MGPB), which are bacteria that enhance the growth of microalgae through several mechanisms [28]. Next, studies will be presented in which a free-living diazotrophic organism, naturally or artificially induced under laboratory conditions, acts as MGPB and improves the growth or certain characteristics of microalgae. This type of interaction has been divided into, on one hand, interactions between microalgae and photosynthetic or non-photosynthetic diazotrophic bacteria, and on the other hand, interactions between microalgae and diazotrophic bacteria in corals and lichens. 

### 2.1. Microalgae Interaction with Non-Photosynthetic Diazotrophic Bacteria

The most numerous studies investigating interactions between microalgae and non-photosynthetic diazotrophic organisms have been conducted on *Azotobacter* spp. and *Azospirillum* spp. Therefore, we will pay special attention to these interactions.

#### 2.1.1. Microalgae Interaction with *Azotobacter* spp.

The obligate aerobe *Azotobacter* sp. is a model organism for studying BNF. *Azotobacter* spp. are organisms that thrive in aerobic conditions. However, they have developed several mechanisms to adapt and carry out strictly anaerobic pathways, such as the BNF. Some of these mechanisms include increased respiration and high exopolysaccharide production [29]. This makes *Azotobacter* an ideal organism to co-culture with microalgae since it does not require growth in anaerobic environments. However, wild-type free-living diazotrophs like *Azotobacter* mainly fix enough N for their own needs and do not excrete significant amounts of fixed N products. In this sense, *Azotobacter* has evolved enzymes and transporters to minimize the loss of fixed N into the surrounding environment [30]. However, under certain conditions, some release of ammonium must occur since studies have shown that the N-fixing capacity of certain *Azotobacter* species (e.g., *A. vinelandii, A. agilis, A. beljerinckii, A. chroococcum*) can support the growth of different microalgal species like *Chlamydomonas* in media with no added N source, solely by using N from the air [31,32]. Indeed, to establish and maintain these co-culture for several years, it was critical to use nitrogen-free and carbon-free media [33]. That is why the absence of carbon must also be essential. Interestingly, the microalgal photosynthetic efficiency was similar to those growing with added N, which strongly suggests that *Azotobacter*, through its N-fixing capacity, provides *Chlamydomonas* with readily available N sources [34]. In return, it is clear that *Chlamydomonas* provides *Azotobacter* with fixed carbon, while *Azotobacter* provides the microalgae with fixed N. However, the precise identity of the exchanged molecules is still unknown, and only indirect clues are available. *Azotobacter* can excrete up to 17 different types of amino acids into the media [33], and *Chlamydomonas* can use some of these amino acids as N source [35]. In this regard, *Chlamydomonas* can also secrete different carbon sources as pyruvate, glycolate, oxalate [36], several keto acids [37], acetate [37], various fermentation products like malic acid, acetic acid, ethanol and formic acid [38] and amino acids and sugars [39]. Some of these compounds are known to be carbon sources for *Azotobacter* and, for this reason, may be the ones chosen for N exchange, although it is unknown which ones in particular.

This *Chlamydomonas-Azotobacter* association was extracellular. However, it has been described that *Azotobacter* and *Chlamydomonas* can fuse their membranes in the presence of polyethylene glycol, which can transform this association from extracellular to endosymbiotic while maintaining the ability to grow in N-deficient medium for several years [40]. In this forced endosymbiotic association between *Chlamydomonas* and *Azotobacter*, the diazotrophic cells were found not only in the *Chlamydomonas* periplasmic space and across the cell wall, but also in organelle-like vesicles located in the *Chlamydomonas* cytoplasm, which resembled true organelles. The number of endosymbiotic bacteria present in the *Chlamydomonas* cytoplasm varied from 1 to 8 per cell, with an average of 1–2 per cell. Also, the number of endosymbiotic bacteria and the volume ratio of *Chlamydomonas-Azotobacter* were kept nearly constant, indicating that these two parameters were critical for stabilizing the interaction [41]. When a third component such as the mycorrhizal fungus *Alternaria* sp. was introduced into this *Chlamydomonas-Azotobacter* consortia, it was observed that the presence of the fungus had a positive impact on the survival of the association in the absence of added N. It has been suggested that the fungus may contribute by releasing sulfur-containing amino acids, such as cystathionine, which could be utilized as a source of sulfur by both the microalgae and the bacteria [42]. 

**Figure 1:**
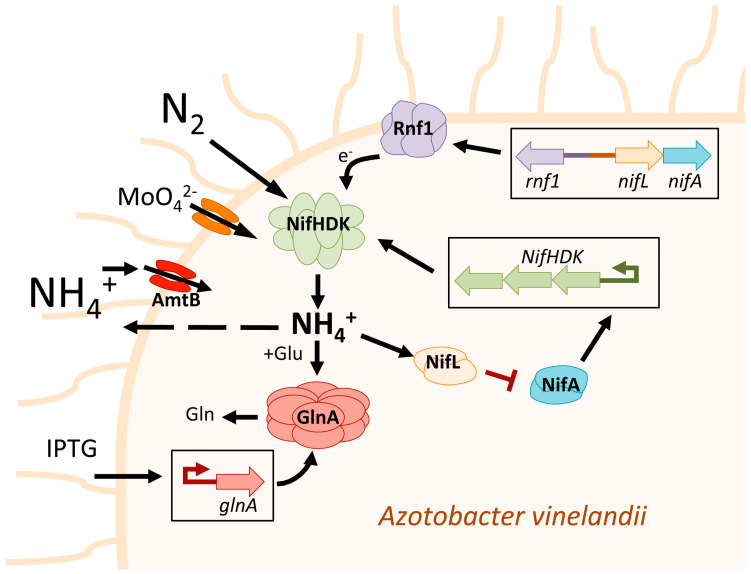
**Schematic representation of the different strategies followed in *Azotobacter vinelandii* to achieve high ammonium excretion**. The operon *NifHDK* refers to a set of three genes, *nifH*, *nifD*, and *nifK*, that encode for the three subunits of nitrogenase. In *Azotobacter vinelandii*, nitrogenase expression is controlled by the two-component operon system *NifL-NifA*, where NifA is an activator and NifL is an anti-activator of nitrogenase. In the *Azotobacter vinelandii AV3* mutant, *NifL* is mutated in a specific manner that prevents the inactivation of NifA by high intracellular ammonium concentration. This mutation leads to constitutive expression of nitrogenase and the release of excess ammonium into the growth medium [43]. Adjacent to operon *NifL*-*NifA* is the operon *rnf1* that is transcribed divergently from *nifLA* promoter. Rnf1 is a membrane-bound complex involved in electron transport to nitrogenase. It is supposed that the increased expression of *rnf1* enhances the electron (e^−^) supply to nitrogenase, thereby increasing its activity and, consequently, the production of ammonium [46]. The increase in molybdate present in the medium is supposed to produce higher molybdate transport, which increases nitrogenase activity and its ammonium production [44,45]. It has been demonstrated that a mutation in the glutamine synthetase gene (*GlnA*) in the *Azotobacter vinelandii AV6* mutant prevents the conversion of ammonium to glutamine, leading to increased ammonium excretion. This is because the ammonium produced by the nitrogenase cannot be incorporated into glutamate (Glu) to form glutamine (Gln) [47]. The *Azotobacter vinelandii AV11* mutant has an inducible *GlnA* promoter that can be activated by IPTG. When IPTG is removed from the medium, *GlnA* is not expressed, and *Azotobacter vinelandii AV11* shows a significant increase in ammonium release [49]. The mutation of the ammonium transporter gene *amtB* prevents the re-entry of excreted ammonium into the cell interior, resulting in an increase in the presence of ammonium in the media [50].

Furthermore, certain strains of *Azotobacter vinelandii* have been genetically modified to secrete a certain amount of fixed N_2_ in the form of ammonium. In *Azotobacter* the nitrogenase expression is controlled by the two-component operon system *NifL-NifA*, where NifA serves as a transcriptional activator for nitrogenase, while NifL acts as an anti-activator by sensing the cell’s redox signals and energy status (Figure 1). The precise deletion of almost the entire *NifL* gene while leaving *NifA* intact (in the *Azotobacter vinelandii AV3* mutant) resulted in constitutive expression of nitrogenase. This mutant presents a deregulated phenotype that produces levels of ammonium higher than the growth requirements of *Azotobacter vinelandii*, resulting in the release of excess ammonium into the growth medium [43] (Figure 1). The microalgae strains *Chlorella sorokiniana RP*, *Pseukirchneriella* sp. *C1D*, and *Scenedesmus obliquus C1S* were able to utilize this ammonium released by the *Azotobacter vinelandii AV3* mutant to grow without requiring any other N source. The nitrogenase is a molybdoenzyme. In this context, the increase in molybdate present in the medium increases the nitrogenase activity and the release of ammonium [44,45] (Figure 1). However, its role in microalgae growth has not been studied yet. Furthermore, studies have shown that the deletion of *NifL* in *Azotobacter vinelandii* accompanied by increased expression of *rnf1*, which encodes an electron transport complex, can enhance the excretion of ammonium by the bacteria, possibly by increasing the availability of reducing equivalents to support nitrogenase catalysis [46] (Figure 1). The amount of carbon source was found to be a critical factor in sustaining high levels of ammonium release by *Azotobacter vinelandii NifL*. It was determined that 2 moles of sucrose were necessary to release one mole of ammonium [45]. 

It has been shown that mutations in the glutamine synthetase *(GlnA*) can also increase the ammonium excretion in *Azotobacter vinelandii* (Figure 1). Specifically, the *Azotobacter vinelandii GlnA* mutant (*AV6*) was found to release a significant amount of ammonium, and the double mutant of *NifL* and *GlnA* (*AV7*) also exhibited considerable ammonium release. The *Azotobacter vinelandii* strains *AV6* and *AV7* supported better growth of the microalgae *Chlorella sorokiniana strain RP* compared to *Azotobacter vinelandii AV3* when atmospheric N_2_ was used as the sole source of nitrogen. This suggests that the increased ammonium excretion resulting from mutations in the *GlnA* and *NifL* genes has a positive impact on the growth of microalgae [47]. These results demonstrate that restricting amino acids synthesis and bacterial biomass through the GlnA inhibition resulted in a more energy-efficient strategy for the biological production of ammonium than deregulating nitrogenase through *NifL* mutation. One of the drawbacks of using *Azotobacter vinelandii GlnA* mutants is their poor growth under diazotrophic conditions. To address this issue, Ambrosio and collaborators developed the *Azotobacter vinelandii AV11* strain, which has an inducible *GlnA* promoter that can be activated by isopropyl-β-thiogalactoside (IPTG) [48] (Figure 1). *Azotobacter vinelandii AV11* requires IPTG to grow diazotrophically, but upon removal of IPTG from the medium, it exhibits a significant release of ammonium, up to 20 mM, which promotes robust growth of the microalgae *S. obliquus* and *Chlorella sorokiniana* [49]. Other approaches have been carried out to increase the production of extracellular ammonium by *Azotobacter vinelandii*. In this regard, the mutation of the ammonium transporter gene *amtB* in *Azotobacter vinelandii* resulted in the production of sufficient levels of extracellular ammonium to support the growth of *Chlorella sorokiniana* [50] (Figure 1). Interestingly, the authors also mutated the urease enzyme system (ureABC). This mutation increased the levels of extracellular urea; however, the concentration released was not sufficient to support the growth of *Chlorella sorokiniana*. 

It has been observed that *Azotobacter* promotes the growth of microalgae using other strategies. In this regard, *Azotobacter vinelandii* in diazotrophic conditions provides the microalgae *Neochloris oleoabundans* and *Scenedesmus* sp. BA03 with azotobactin, a peptide siderophore, as a N source to support their growth [51]. This interaction appears to be commensalistic since *Azotobacter vinelandii* is apparently not benefiting from the microalgae. One of the challenges with using *Azotobacter vinelandii* mutants that accumulate ammonium is that any mutation that improves the growth rate while no longer fixing nitrogen will result in a subpopulation of cheaters, taking advantage of the accumulated ammonium. In this sense, some laboratories have reported that the mutation in the *NifL* gene is not very stable and over time spontaneous populations of revertants appear [49,52]. A question that needs to be resolved, especially if it is intended to cultivate these strains of *Azotobacter* and microalgae for a long time.

#### 2.1.2. Microalgae Interaction with *Azospirillum* spp.

The free-living diazotrophs belonging to the *Azospirillum* genus are a group of plant rhizosphere bacteria that have been widely recognized as model PGPB due to their positive impact on plant growth, crop yields, and nitrogen content [53]. Bacteria belonging to this genus synthesize one polar flagellum during growth in liquid medium, primarily used for swimming towards optimal oxygen concentrations, a behavior known as aerotaxis [54]. *Azospirillum* does not excrete significant amounts of ammonium under diazotrophic growth. Therefore, in addition to biological nitrogen fixation, its plant stimulatory effect has also been attributed to several other mechanisms, which are reviewed in detail in [55]. Among these mechanisms, several stand out, including the secretion of the auxin phytohormone indole-3-acetic acid (IAA) [56], polyamine and trehalose production, an increase in the rate of mineral uptake by the plant [57], and the production of bioactive volatile organics, such as 2,3-butanediol and acetoin [58]. Apart from its well-known interaction with plants, several studies have also shown that *Azospirillum* is capable of establishing different interactions with microalgae mainly due to its auxin-production capacity [59]. In this regard, it has been described that *Azospirillum brasilense* in consortia with *Chlorella* species could almost completely mitigate the oxidative stress in the microalgae derived from N limitation [60], enhance removal of ammonium and phosphorus in wastewater [61], and increase the ammonium [62], polyphosphate [63], starch [64,65] and the fatty acid intracellular accumulation of *Chlorella* [66]. It has also been studied that *Azospirillum brasilense* maintains a mutualistic interaction with the microalgae *Scenedesmus obliquus*, *Chlorella vulgaris*, and *Chlamydomonas reinhardtii*, supported by the exchange of IAA and tryptophan, respectively. This interaction increases the microalgal CO_2_ fixation [67]. In addition to tryptophan, it has been described that thiamine released by *Chlorella sorokiniana* also plays a role in mutualism with *Azospirillum brasilense* [68]. 

It has been described that the N fixation by *Azospirillum* only occurs under microaerophilic conditions [69] which are unlikely to exist in the hyper-oxygenated environment of a microalgae culture. For this reason, its N-fixing capacity has not been considered important in any of the interactions between *Azospirillum* and microalgae described so far. However, some of these studies have been performed by co-immobilizing the microalgae *C. vulgaris* and *Chlorella sorokiniana* with *Azospirillum brasilense* in alginate beads [61,70,71,72,73,74]. Light microscopy revealed that both microorganisms colonized the same cavities inside the beads [75]. Moreover, it has been described that the interior of large alginate beads may become oxygen deficient [76]. Therefore, it is possible that in these microaerophilic spaces, *Azospirillum* could be fixing nitrogen and releasing it into the bead where the microalgae reside. As a result, the nitrogen fixation carried out by *Azospirillum* may be contributing more to these interactions than previously believed. Further experiments to test this hypothesis would be highly valuable.

#### 2.1.3. Microalgae Interaction with Other Diazotrophic Bacteria

Apart from *Azotobacter* and *Azospirillum*, few diazotrophic bacterial have been studied for their interactions with microalgae. Co-culturing *Chlorella vulgaris* with the N_2_ fixing bacteria *Bacillus pumilus ES4* has been shown to enhance the growth of microalgae, likely due to the N fixing ability of *Bacillus pumilus*, which leads to the accumulation of ammonium in the medium. This growth increase is noticeable only when no N is added to the media, suggesting that the microalgae may be assimilating some of the N fixed by *Bacillus pumilus* [77]. *Nannochloropsis oceanica* is a marine microalga known for its high production of eicosapentaenoic acid (EPA), making it one of the most commonly cultivated microalgae for aquaculture and food supplements. In a study, 10 bacteria were isolated and co-cultivated with *Nannochloropsis oceanica*, resulting in increased biomass and EPA production. The analysis showed that out of the bacteria tested, seven were capable of fixing N. Among them, *Nitratireductor* sp., a diazotrophic bacterium, caused the greatest increase in biomass [78]. 

### 2.2. Microalgae Interaction with Photosynthetic Diazotrophic Bacteria

*Cereibacter sphaeroides* (previously known as *Rhodobacter sphaeroides*) is an aerobic, photosynthetic [79] nitrogen fixing bacterium that secretes polysaccharides to avoid inactivation of nitrogenase, which leads to a decrease in the partial pressure of oxygen [80]. *Cereibacter sphaeroides* and the microalga *Coelastrella* sp. KGU-HN001 have been shown to maintain a mutualistic exchange that allows both organisms to grow in media with air as the only source of nitrogen and carbon. In the coculture, *Coelastrella* secreted several saccharides, and the authors propose that maltose is likely the carbon source used by *Cereibacter sphaeroides* [81]. Cyanobacteria is a wide and diversified phylum of photosynthetic bacteria. Some of them are capable of both oxygenic photosynthesis and N fixation. However, their N-fixing enzyme, the nitrogenase, is sensitive to oxygen [82]. Therefore, these organisms usually separate N fixation from photosynthesis in specialized cells called heterocysts. Heterocysts provide cyanobacteria vegetative cells with fixed N. In return, the vegetative cells provide the heterocysts with reduced carbon through photosynthesis, which is needed to support N fixation [83]. One of the characteristics of cyanobacteria, especially those forming heterocysts, is their high propensity for symbioses. Cyanobacteria establish various types of symbiosis with the host with variable degrees of integration. Most of these interactions have been described with terrestrial plants but also with hornworts, liverworts, ferns, cycads, cyanolichens, and angiosperms [84]. Additionally, interactions between N fixing cyanobacteria and microalgae, particularly diatoms (*Bacillariophyceae*), have been described. Diatoms are an ecologically and morphologically diverse group of microalgae that have a cell wall that incorporates silica as a main component. These interactions are called diatom-diazotroph associations (DDAs) and provide an essential contribution to oceanic CO_2_ fixation (primary productivity) supported by N fixation [85]. In DDAs, symbiotic diazotrophs form trichomes where one cell is specialized in N fixation, the heterocyst, while the remaining cells, the vegetative cells, are phototrophic and divide, whereas heterocysts cannot [86]. In marine environment trichomes receive essential nutrients that allow them to grow more efficiently in symbiosis [87], being unable to grow in the laboratory [88]. Unlike terrestrial systems, the interaction between marine diatoms and cyanobacteria is less characterized. In the DDAs of the diatom microalgae *Hemiaulus hauckii* and heterocystous cyanobacteria *Richelia intracellularis*, it has been shown that carbon transfer from the diatom enables faster growth and N fixation rate by the trichomes. The authors discovered that N fixation is 5.5 times higher during symbiosis, and that 25% of the fixed carbon from the host diatom is transferred to the symbiotic trichomes to support the high rate of N fixation. In turn, 82% of the N fixed by the trichomes ends up in the host. *Richelia intracellularis* can be located inside the cytoplasm of *Hemiaulus hauckii*, either partially (between the diatom plasma membrane and the cell wall) or completely external. Moreover, this location is related to the age of the association, with the older symbionts being more internal [89]. The associations of the diatoms *Rhizosolenia Brightwell* and *Hemiaulus Ehrenberg*, with *Richelia intracellularis* is hypothesized to have a critical role in the development of microalgae blooms in N limited region [90]. 

Measurements of nitrogen and carbon fixation rates have been reported for symbiotic associations of microalgae and cyanobacteria in oceans, but the majority of these measurements are based on bulk or plankton cell concentrates [91,92]. However, there are studies conducted in oligotrophic oceans where the rate of N fixation by symbiotic filamentous cyanobacteria *Richelia intracellularis* and *Calothrix rhizosoleniae*, and the transfer of N to their diatom partners *(Hemiaulus* spp., *Rhizosolenia* spp., and *Chaetoceros compressus*) have been measured [93]. The N fixation rates calculated for *Calothrix* and *Richelia* symbionts were 171–420 times higher when the cells were in symbiosis with the diatoms, compared to the rates estimated for the cells living independently. Furthermore, up to 97.3% of the fixed N is transferred to the diatom partners, and not assimilated by the symbionts themselves, nor released into the environment. Interestingly, having more trichomes in a single host diatom reduces the demand for N fixation per trichome, thereby decreasing their cost of carbon [87]. In agreement with that the diatom partners also have a positive influence on the metabolism and growth of their cyanobacterial symbionts [93]. In another study, also involving the diatom-*Richelia* system, the authors investigated how the partners coordinate their carbon fixation and how the diatom ensures that *Richelia* maintains a high rate of N fixation well above its needs. To achieve this, the researchers inhibited diatom photosynthesis with inhibitors, resulting in a decrease in the N fixation rate of *Richelia*. These findings suggest that despite the *Richelia* ability to fix their own carbon, it still relies on the diatoms for carbon fixation. This is likely because *Richelia* requires an additional supply of carbon to support the increased N fixation, which is provided by the photosynthetic activity of the diatom. Supporting this idea, up to 22% of the carbon assimilated by *Richelia* comes from the diatom [94].

As mentioned earlier, the heterocyst-forming cyanobacterium *Richelia intracellularis* provides fixed N to the diatom *Hemiaulus hauckii*, and hence there must also be mechanism(s) for molecular transfer of fixed N from the cyanobacterium to the diatom. To study this, an electron microscopy analysis was performed on the relationship between *Hemiaulus hauckii* and *Richelia intracellularis*, which revealed that the filaments of the *Richelia intracellularis* symbiont, typically composed of one terminal heterocyst and three or four vegetative cells, are located in the diatom’s cytoplasm and are not enclosed by a host membrane [95]. Interestingly, numerous membrane vesicles were detected in the vegetative cells of *Richelia intracellularis*. These vesicles can export cytoplasmic material from the bacterium, leading the authors to suggest that they represent a vehicle for the transfer of fixed N from *Richelia intracellularis* to the diatom’s cytoplasm. It is worth noting that the genome of *Richelia intracellularis*, which is 3.2 Mbp, is much smaller than the genome of most free-living heterocyst-forming cyanobacteria, which is typically 7–9 Mbp [96]. Therefore, the authors hypothesize that these vesicles, apart from transporting N, could also serve as a vehicle for gene transfer. Additionally, the authors observed a possible association of the cyanobacteria with the mitochondria of *Hemiaulus hauckii* and proposed that this association may be important for protecting the N-fixing enzyme, nitrogenase, from oxygen produced during photosynthesis. The oxygen-respiring mitochondria would help to decrease the level of intracellular oxygen in the vicinity of the cyanobacterium [95].

The amount and chemical form (ammonium, amino acids, etc.) in which N is transferred in the DDAs is unknown, as well as the role of the host in potentially providing glutamate or other C skeletons to the symbionts, and its influence on the N-fixation in the symbiont. Certain transporters encoded in the genomes of cyanobacteria have been proposed as responsible for transferring specific compounds between the symbiont and host. To investigate this, membrane transporters have been studied in three different types of DDAs according to the cellular location of the symbiont: the *Hemiaulus hauckii- Richelia intracellularis* RintHH01 symbiosis (internal association), *Rhizosolenia clevei- Richelia intracellularis* RintHM0 (partial association), and *Chaetoceros compresus-Calothrix rhizosoleniae* CalSC01 (external association) [97]. The truly internal *Richelia* symbionts of *Hemiaulus* spp. are proposed to be renamed as *Richelia euintracellularis* and the externally attached symbionts of *Chaetoceros compressus* are removed from the genus *Calothrix* and renamed *Richelia rhizosoleniae* [98]. When the symbiont is internal or partially internal, it contains a similar array of transporters. In contrast, the external symbiont has transporters that are similar to those of endosymbionts, as well as other transporters that are useful for life in a free-living form. The authors hypothesized that glutamine and arginine are transferred from the symbiont to the host in these DDAs, but the presence of ammonium transporter proteins, specifically in *Calothrix rhizosoleniae* CalSC01, and the presence of the NKCC1-type cation transporter in all three symbionts, suggest that ammonium may be a possible N vehicle in at least some DDAs.

The symbiotic association between three N-fixing cyanobacteria *Anabaena variabilis*, *Westiellopsis prolifica* and *Nostoc muscorum* with three green microalgae *Chlorella vulgaris*, *Scenedesmus obliquus* and *Botryococcus braunii* was studied under N-deficient conditions. Out of the nine interactions studied, the synergism between *Botryococcus braunii* and *Nostoc muscorum* was the best established, with a 50% enhancement in N fixation in co-culture. Analysis of their secretome revealed the presence of new secondary metabolites that have roles in quorum signaling, carbon metabolism, N fixation, lipid metabolism, and antimicrobial activity [99]. Some of these compounds may have interesting roles, such as 9-octadecenamide, a known phytohormone [100] and 1,30-triacontanediol, which improves glucose-lipid metabolism and exhibits antimicrobial activity [101].

A small number of marine microalgae have acquired cyanobacterial endosymbionts with the ability to fix nitrogen, allowing them to thrive in nitrogen-limited environments. These microalgae harbor the N-fixing cyanobacteria as organelles of endosymbiotic origin, known as spheroid bodies. Recently, these spheroid bodies have been propose to be renamed as ‘diazoplasts’ [102]. These organelles are distinctive from all other unicellular N-fixing cyanobacteria in that they only fix N in the presence of light [103]. The diazoplasts encode genes for N fixation and have the capacity to fix molecular N_2_, but they have lost the ability to perform photosynthesis. These organelles have been identified in the diatom microalga *Rhopalodia gibba*, being the closest free-living relatives diazotrophic cyanobacteria of the *Cyanothece* sp. group [103]. The unicellular cyanobacteria *Crocosphaera* spp. [104] appears to be highly favored for this type of endosymbiotic interaction. In fact, permanent endosymbionts that are closely related to the *Crocosphaera* genus, particularly *Crocosphaera subtropica*, are found in various microalgae including the diatoms *Epithemia* spp. and *Climacodium frauenfeldianum* [105], as well as the coccolithophore *Braarudosphaera bigelowii* [106]. Despite the diverse morphology of *Epithemia* spp., it is believed that their N-fixing endosymbiont originate from a solitary endosymbiotic event with a cyanobacteria belonging to the *Crocosphaera* genus [107]. The carbohydrate catabolism in the diazoplast implies that both the oxidative pentose pathway and oxidative phosphorylation work together to produce ATP and reducing equivalents, while consuming oxygen to facilitate the nitrogenase activity. The diazoplast has a reduced capacity to utilize alternate sources of N, in contrast to its increased nitrogenase activity [102].

Other example studied is the endosymbiotic cyanobacterium *Candidatus Atelocyanobacterium thalassa* (UCYN-A), which are obligate endosymbionts of single-celled haptophyte microalgae [108,109]. It has been described that UCYN-A has an endosymbiotic association with several picoeukaryotic microalgae of the *Prymnesiophyceae* class such as *Emiliania huxleyi*. The partnership is mutualistic because *Emiliania. huxleyi* receive fixed N from UCYN-A, and in exchange, transfer fixed carbon to UCYN-A [110]. It has been characterized that UCYN-A also establishes an endosymbiotic relationship with the microalga *Braarudosphaera bigelowii* [106]. Another interaction studied is the endosymbiotic relationship in marine environments between cyanobacteria of the UCYN-C group, closely related to *Crocosphaera*, and two diatoms from the *Rhopalodiaceae* family, *Epithemia pelagica* sp. and *Epithemia catenata* sp. These symbioses were challenging to detect because the endosymbionts lack fluorescent of the pigments involved in photosynthesis, have nitrogenase gene sequence similar to free-living cyanobacteria, and are only formed in N-deficient media [111]. Interestingly, it has recently been demonstrated in the oceans that species belonging to the UCYN groups are significant exporters of organic matter in the form of nitrogen and carbon, despite their small size, which is fixed in the deep sea. In this study, UCYN-B and UCYN-C cells were found to be embedded in large organic aggregates comprised of tens to hundreds of cells connected by an extracellular matrix, presumably facilitating this process [112].

### 2.3. Microalgae Interaction with Diazotrophic Bacteria in the Corals

Corals provide a habitat for many different organisms and are considered as multipartite symbiotic organisms, or holobionts, formed by the animal host (*Cnidarians*) and a diverse microbiota consisting mainly of microalgae from the genus *Symbiodinium*, as well as bacteria, archaea, fungi, and viruses [113]. The symbiotic association between the coral host and photoautotrophic microalgae of the genus *Symbiodinium*, commonly referred to as zooxanthellae, has been well studied in corals, see reviews [114,115]. These microalgae commonly reside in the endoderm of corals, where the coral host provides inorganic nutrients in exchange for photosynthetically fixed carbon and amino acids. Nitrogen assimilation is of particular importance for corals living in oligotrophic reef waters, as it is a major factor that limits their net primary productivity. Interestingly, N fixation activity have been detected in corals [116] and diazotrophic organism have been identified as their main responsible [117,118]. The coral’s acquisition of N through this process is called diazotroph-derived nitrogen (DDN). In this sense, a wide variety of cyanobacterial and non-cyanobacterial diazotrophic communities have been discovered in coral tissue, within the surface mucus layer, or in the coral skeleton [119]. These diazotrophic organisms, such as *Cyanothece*, have been shown to be taken up by the corals from the surrounding seawater [120,121]. The dominant diazotrophic organisms in corals are closely related to the bacterial group rhizobia, therefore it has been hypothesized that, as in terrestrial plants, these diazotrophs have developed a mutualistic relationship with corals and contribute to fixing N for the microalgae *Symbiodinium* [122]. Interestingly, It has been found that in *Montipora* corals, the abundances of *Symbiodinium* and diazotrophic bacteria closely related to the *Vibrio* genus were positively correlated [123].

It has been shown that the DDN is assimilated not only by the animal host but also by *Symbiodinium*, as highlighted in the review [124]. In relation to that, experiments have shown that in tropical corals such as *Pocillopora damicornis* and *Stylophora pistillata* as well as in Hawaiian corals of the genus *Montipora*, DDN notably increases the N enrichment of microalgae *Symbiodinium* [120,125]. This suggests that diazotrophs are an important source of N for the microalgae and increase the standing stock of their population. Interestingly, DDN has been linked to the ability of corals to deal with different types of stress. In this sense, it has been shown that DDN could trigger coral bleaching by generating an imbalanced nutrient supply, leading to phosphorus starvation of *Symbiodinium* [126]. During heat stress, the enrichment of dissolved organic carbon increases the abundances of diazotrophs in *Xenia umbellata* and *Pinnigorgia flava* corals, which increases N availability and destabilizes the coral-algal symbiosis [127]. However, beneficial effects of DDN in coral bleaching have also been reported. In the coral *Oculina Patagonica*, DDN has been described to facilitate the rapid proliferation of microalgae, which provide an alternative carbon source for bleached corals, helping them to recover [128]. DDN has been shown to increase as a mechanism for the coral to deal with thermal stress [129]. However, whether this N input is beneficial or not for the microalgae-host symbiotic association to deal with thermal increase remains to be resolved.

### 2.4. Microalgae Interaction with Diazotrophic Bacteria in the Lichens

Lichens are regarded as highly successful life forms due to their self-sustaining ecosystems that involve a diverse microbiota comprising various kingdoms of life in intricate, albeit not fully comprehended, associations [130,131]. Lichens are fascinating organisms in which a fungus (mycobiont) lives in an intimate relationship with at least one photosynthetic organism (photobiont). The photosynthetic organism in lichens can be either a microalgae or a filamentous alga (known as chlorolichens), a cyanobacterium (cyanolichens), or both (known as tripartite lichens or photosymbiodemes) [132,133]. Although lichens are generally defined as bipartite or tripartite associations, a wide array of bacterial communities are also present in a stable and host-specific manner. However, the precise role of these bacteria is still unknown [134]. The presence of these bacteria has been linked to the difficulty of resynthesizing or recreating some lichens under laboratory conditions through the coculture of their fungal and algal partners [135]. The microalgae *Trebouxia*, *Coccomyxa* and the filamentous alga *Trentepohlia* are the most commonly recruited green microalgae that form symbiotic relationships with the mycobiont [136]. In the context of lichen symbiosis, while there is a wealth of information on the interaction between the fungus and the photosynthetic partner, there has been relatively little research on the relationship between the microalgae and cyanobacteria in tripartite lichens. This stable symbiotic relationship enables chlorolichens to be C-autotrophs and cyanolichens to be C-autotrophs and N-autotrophs. These characteristics enable lichens to colonize nutrient-poor areas [137]. In cyanolichens, the cyanobacterium is referred to as the cyanobiont. In tripartite lichens, the cyanobiont is mainly responsible for N fixation [138] and it has been observed that lichen fitness increases through the specialization of the cyanobiont in nitrogen fixation [139]. To prevent the inhibition of nitrogenase by oxygen, the fungus partner of the lichen creates an environment with reduced oxygen levels and harbors the cyanobiont cells within internal structures called cephalodia, which are gall-like in appearance [140].

The cyanobiont in lichens is typically filamentous and capable of forming heterocysts (e.g., *Calothrix*, *Nostoc*). Nitrogen fixation is beneficial not only to the cyanobiont but also to both the fungal and, interestingly, the algal partners. The N-fixation capacity of the cyanobiont strongly and positively correlates with the N content and the maximum photosynthetic rate of the green alga [141]. It has been observed that the cyanobiont is dependent on the algal population for the majority of its carbon income [142]. Furthermore, this dependency is stronger in lichens with internal cephalodia such as *Placopsis pycnotheca*, which have lower exposure to light [143]. It should be noted that a direct physical interaction between cyanobacteria and green microalgae has never been observed [144]. Interestingly, the frequency of heterocysts respect to the total cells in bipartite lichens varies between 2–8%, whereas in tripartite lichens it varies between 10–55%, with higher rates of N fixation [139]. Nevertheless, in tripartite lichens, the number of cyanobionts is always kept lower than that of the algal photobiont, although the reason for this is unknown. Microalgae could potentially receive multiple benefits from this fixed N. Cyanolichens show higher concentrations of organic N compared to chlorolichens. Therefore, it has been concluded that cyanolichens may have an advantage in inhabiting special ecological niches, such as extremely oligotrophic habitats [145]. It is worth noting that the association with diazotrophic cyanobacteria has allowed lichens to surpass size limitations in oligotrophic environments [146].

It has been described that non-cyanobacterial diazotrophs are also present and are capable of performing N-fixation in association with lichens, particularly *Azotobacter* spp., *Actinomycetes* or lichen-associated *Rhizobiales* [147,148,149,150]. However, studies investigating the relationship between these non-cyanobacterial diazotrophs and the photobiont green microalgae are limited. Tripartite lichens have been found to synthesize a unique class of chemicals known as mycosporine-like amino acids [151], but their involvement in the association with the green microalgae requires further investigation.

## 3. Biotechnological Potential

The microalgae have a huge metabolic diversity, fast growth, and low production costs, do not compete for arable lands, can be cultivated throughout the year, and usually are more productive per unit of land area than any plant system, characteristics that make these organisms very useful in biotechnological applications aimed at meeting urgent needs in industry and agriculture [152]. Cyanobacteria also possess a series of favorable characteristics that have led them to be widely used in various biotechnological applications, as highlighted in the review [153]. For instance, microalgae are used in wastewater treatment, as sustainable sources to produce high-value compounds, fertilizers, hydrogen, biofuel, or livestock feed, as well as a way of carbon sequestration to avoid CO_2_ increase and global warming [154]. Microalgae possess a carbon fixation capacity that is 10–50 times higher than that of terrestrial plants [155]. In this regard, the potential of microalgae to mitigate global warming by reducing atmospheric CO_2_ through carbon sequestration, based on their rapid growth rate and biomass production, has been reviewed [156]. To enhance this carbon fixation capacity, an increase in N supply is necessary to provide the N required for nucleotide or protein biosynthesis, among other functions. Nevertheless, from a biotechnological point of view, there are still some inefficient procedures that can be improve by means of their interaction with other microorganisms, mainly bacteria and great efforts are being made to improve them [157]. 

**Figure 2:**
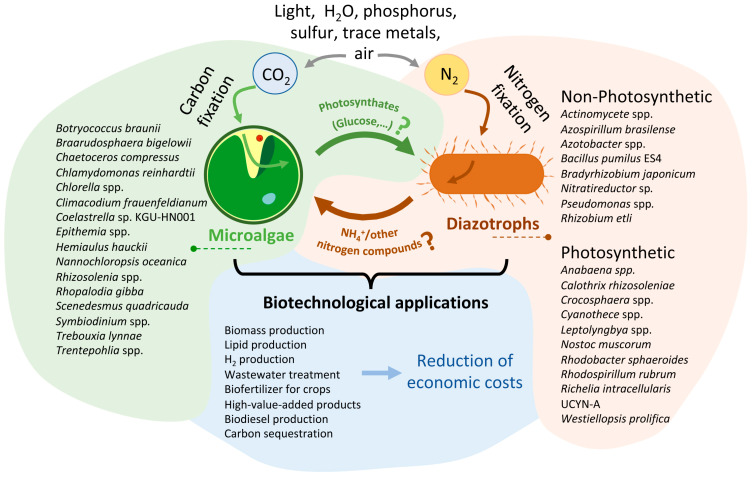
**Conceptual diagram of the different associations and biotechnological application between microalgae and diazotrophic organisms presented in this review**. This figure represents a theoretical model of a carbon-nitrogen exchange system that utilizes air, light, water, phosphorus, sulfur, and trace metals to produce biomass and other biotechnological products. On the left, there is a drawing showing a microalga, and on the right, another drawing representing a diazotrophic organism. Diazotrophic photosynthetic organisms can also fix CO_2_, so there should be an arrow indicating this fact. However, to ensure that the figure clearly conveys the intended message, this fact is indicated here. The names of the main species of organisms described in this article are illustrated next to each organism. Next to the arrows, the main molecules exchanged during the interaction between both microorganisms are indicated. As indicated in the manuscript, the compounds exchanged between the two microorganisms have been more numerous (hormones as IAA, vitamins, etc.). However, in the figure, only those produced directly from the fixation of CO_2_ (photosynthates) and N_2_ (ammonium or other nitrogen compound) are represented. The question mark indicates that, as described in the article, in many cases, the specific photosynthate released is unknown. Additionally, not all of the diazotroph species mentioned have been reported to release ammonia or other N sources; for some of them, this is merely a hypothesis. The bottom part of the figure shows the main biotechnological applications described for these consortia. In the future, genetic manipulation engineering could significantly reduce the economic cost of producing such compounds through these consortia. For more details, refer to the text.

Many of the biotechnological applications of microalgae rely on axenic cultures, but numerous strains of microalgae isolated from nature cannot be grown under axenic laboratory conditions. This means that these strains have become dependent on other organisms due to persistent interactions in their natural habitat. This reliance on other microorganisms is likely due to their long-term co-evolution [158]. Moreover, axenic cultures are more susceptible to contamination, predation, and collapse. Thus, the utilization of co-cultures consisting of multiple species that can adapt to specific conditions could solve this problem [157]. Open ponds are more prone to contamination; however, they are more cost-effective and easier to build than enclosed photobioreactors, so they are preferred for large-scale microalgae cultivation. A scrutiny has been carried out to see which bacterium increase the biomass production of the microalga *Chlorella sorokiniana* in open ponds. Interestingly, the bacterium that produced the best results was the N-fixing *Rhizobium* sp. [159]. Thus, it is possible that the N-fixing capacity of *Rhizobium* sp. provides a selective advantage to microalgae in open ponds. More studies are needed to corroborate this hypothesis, which would improve the cost of microalgae production for many biotechnological applications (Figure 2).

The use of microalgae biomass as a source of biofuel has been explored extensively [8]. However, the high cost of N fertilizers used in the cultivation process remains a major challenge. Eutrophication caused by the abusive use of chemical fertilizers has a huge environmental impacts and therefore is a current global problem [160]. Microalgae cocultivation with diazotrophic organisms has been seen to increase biomass and lipid production. In this sense, *Azotobacter chroococcum* can simultaneously enhance lipid and biomass productivity by promoting growth and inducing regulatory changes in the lipid metabolism of *Chlamydomonas* [161]. A study found that the co-culture of *Chlorella vulgaris* with *Azotobacter Mesorhizobium* sp. in N-deficient conditions increased the biomass and lipid of *Chlorella vulgaris* [162]. Growth, lipid, and biomass productivity of *C. vulgaris* increased remarkably in coculture with the cyanobacteria *Actinomycet* sp. [163], however there is no data about nitrogenase activity in this study, therefore the growth promotion could also be attributed to their production of phytohormones and vitamins [164]. Although it cannot be ruled out that increased growth could also be explained by the presence of N-rich bacterial debris in the system. Based on these data, it can be concluded that the symbiosis between N-fixing organisms and microalgae can be considered an alternative strategy for mass cultivation of microalgae with low N costs, for sustainable production of next-generation biofuels (Figure 2).

Hydrogen production by microorganisms is an area of active research, and there are several different approaches being explored. One approach is to use microorganisms that are capable of fermenting organic matter to produce H_2_. Some of these microorganisms are diazotrophic. In this regard, it has been observed that the utilization of microalgae, such as *Chlamydomonas*, can improve the efficiency of H_2_ production by different diazotrophic species, such as *Cereibacter sphaeroides* KD131 and *Rhodospirillum rubrum* [165,166]. On the other hand, some microalgae are capable of producing H_2_ by themselves, and one of the best-studied examples is *Chlamydomonas*. In this regard, it has been shown that H_2_ production by *Chlamydomonas* can be improved in the presence of certain diazotrophic bacteria. These bacteria include, among others, the soil bacteria *Pseudomonas stutzeri*, *Rhizobium etli*, *Pseudomonas fluorescens*, *Azotobacter chroococcum*, *Mesorhizobium sangaii*, *Bradyrhizobium japonicum,* see reviews [9,167]. However, until now, it has not been studied in detail whether this increased production of H_2_ is related to N fixation.

Bacteria are widely used in wastewater treatment; however, this has at least two drawbacks, they release the greenhouse gas CO_2_ and need large O_2_ supply which make this procedure not environmentally friendly and economically expensive. Therefore, the co-cultivation with oxygen-producing and CO_2_ fixating microalgae has become a promising way to eliminate those inconveniences [168]. However, studies focusing on the role of N-fixing organisms in these consortia are scarce. In a study, it has been shown that the N-fixing bacteria *Azotobacter beijerinckii* enhanced the biomass production and improved the wastewater treatment in co-culture with *Auxenochlorella pyrenoidosa* [169].

The green biotechnology in agriculture is based on the use of microalgae biomass as soil biofertilizer for crop cultivation and phytoremediation, reviewed in [170]. Furthermore, there is increasing interest in the use of N-fixing bacteria as a biofertilizer for crops [18]. Free-living aerobic diazotroph like *Azotobacter* spp. has been widely used as plant biofertilizer, successfully increasing the yields of a wide variety of crop plants [20]. Combining both strategies, the association of microalgae with N-fixing bacteria, can provide a sustainable solution by reducing the cost and environmental impact of fertilizers (Figure 2). However, the studies investigating whether the consortium of microalgae and N-fixing bacteria enhances this action are limited. It has been described that the diazotrophic cyanobacterium *Anabaena azotica* in co-culture with *Auxenochlorella pyrenoidosa* can be used as biofertilizers increasing the content of unsaturated fatty acid, and improve the content of nutrients in the fruits of *Crataegi fructus* [171]. Although more studies are needed to validate these results, inducing symbiosis between microalgae and N-fixing bacteria may have great potential in the next generation of biofertilizers in agriculture.

## 4. Concluding Remarks

Microalgae-based products have not been extensively developed due to the high costs associated with microalgae harvesting, maintenance, and extraction of their compounds. One of the biggest limitations is the economic cost of the N source used in these cultures. This review summarizes studies that investigate associations between microalgae and N-fixing organisms, whether induced artificially in the laboratory or naturally present in the environment. Overall, it can be concluded that the mechanisms mediating these interactions are increasingly being understood, and the more we learn, the closer we come to being able to cultivate microalgae exclusively using the N supplied by these organisms. This would result in a significant reduction in production costs. As mentioned, microalgae, through photosynthesis, fix atmospheric CO_2_ into carbon compounds or photosynthates. Another field in which future research could improve the economic profitability of such associations would be to enable a portion of these photosynthates produced by the microalgae to be released and donated to the diazotrophic bacteria in exchange for the N fixed by them. This would result in co-cultures of microalgae and diazotrophic bacteria using air as the sole source of carbon and nitrogen. Microalgae widely used as model organisms such as *Chlamydomonas* and *Chlorella*, along with aerobic diazotrophic bacteria such as *Azotobacter*, appear to be the best positioned for future research to achieve this goal.

## Data Availability

All data required to evaluate the conclusions of this paper are included in the main text.
